# Diversity and evolution of cytochrome P450s of *Jacobaea vulgaris* and *Jacobaea aquatica*

**DOI:** 10.1186/s12870-020-02532-y

**Published:** 2020-07-20

**Authors:** Yangan Chen, Peter G. L. Klinkhamer, Johan Memelink, Klaas Vrieling

**Affiliations:** 1 Plant Ecology and Phytochemistry, Institute of Biology, Leiden University, Sylviusweg 72, P. O. Box 9505, 2300 RA Leiden, The Netherlands; 2 Plant Cell Physiology, Institute of Biology, Leiden University, Sylviusweg 72, P. O. Box 9505, 2300 RA Leiden, The Netherlands

**Keywords:** Chemical diversity, Pyrrolizidine alkaloid biosynthesis, RNA-seq, Conserved motifs, Phylogeny

## Abstract

**Background:**

Collectively, plants produce a huge variety of secondary metabolites (SMs) which are involved in the adaptation of plants to biotic and abiotic stresses. The most characteristic feature of SMs is their striking inter- and intraspecific chemical diversity. Cytochrome P450 monooxygenases (CYPs) often play an important role in the biosynthesis of SMs and thus in the evolution of chemical diversity. Here we studied the diversity and evolution of CYPs of two *Jacobaea* species which contain a characteristic group of SMs namely the pyrrolizidine alkaloids (PAs).

**Results:**

We retrieved CYPs from RNA-seq data of *J. vulgaris* and *J. aquatica*, resulting in 221 and 157 full-length CYP genes, respectively. The analyses of conserved motifs confirmed that *Jacobaea* CYP proteins share conserved motifs including the heme-binding signature, the PERF motif, the K-helix and the I-helix. KEGG annotation revealed that the CYPs assigned as being SM metabolic pathway genes were all from the CYP71 clan but no CYPs were assigned as being involved in alkaloid pathways. Phylogenetic analyses of full-length CYPs were conducted for the six largest CYP families of *Jacobaea* (CYP71, CYP76, CYP706, CYP82, CYP93 and CYP72) and were compared with CYPs of two other members of the Asteraceae, *Helianthus annuus* and *Lactuca sativa*, and with *Arabidopsis thaliana*. The phylogenetic trees showed strong lineage specific diversification of CYPs, implying that the evolution of CYPs has been very fast even within the Asteraceae family. Only in the closely related species *J. vulgaris* and *J. aquatica*, CYPs were found often in pairs, confirming a close relationship in the evolutionary history.

**Conclusions:**

This study discovered 378 full-length CYPs in *Jacobaea* species, which can be used for future exploration of their functions, including possible involvement in PA biosynthesis and PA diversity.

## Background

Plants produce a great variety of secondary metabolites (SMs) which are involved in the adaptation of plants to both biotic and abiotic stresses [1–3]. At present, more than 200,000 SMs have been isolated and identified, including different chemical classes such as glucosinolates, alkaloids, terpenes, and flavonoids. Typically, species within a clade share similar classes of SMs [2]. For example, glucosinolates are major SMs near-universally in the Brassicaceae, the Capparidaeae and the Caricaceae [4], and benzylisoquinoline alkaloids occur mainly in the Papaveraceae, the Ranunculaceae, the Berberidaceae and the Menispermaceae [5], while pyrrolizidine alkaloids (PAs) distribute preferably in the Asteraceae, the Boraginaceae, the Fabaceae and the Orchidaceae families [6]. Each class of SMs contains a number of similar molecules derived from the same skeleton mostly differing in substitution groups by addition of a number of polar and non-polar substituents. This structural diversity is well documented for PAs in *Jacobaea* species in the Asteraceae family. Thirty-seven structurally related PAs have been detected in *Jacobaea vulgaris* Gaertn*.*, *Jacobaea aquatica* (Hill) G.Gaertn., B.Mey. & Scherb and their hybrids [7]. As yet, it is not fully understood how secondary metabolite diversity comes about and why it is maintained in nature.

To understand the origin of SM diversity, molecular investigations of SM biosynthetic pathways are promising as it is believed that SM diversity of plants is under genetic control [8–11]. Genes involved in SM biosynthesis have often evolved from genes involved in primary metabolism by gene duplication with successive diversification [4, 12]. Many of these genes involved in SM pathways belong to large gene families [3], such as cytochrome P450s [13, 14]. Cytochrome P450 monooxygenase (CYP) genes form a large family in any given plant species and play important roles in secondary metabolism [15]. Many CYPs are involved in biosynthesis of various SMs as they catalyze the oxidative modifications of various substrates using oxygen and NAD(P)H. Structurally, all plant CYPs found so far are membrane-bound enzymes and are mainly anchored in the endoplasmic reticulum membrane via a hydrophobic signal sequence at the N-terminus [16, 17]. CYP proteins share well-conserved motifs including the heme-binding signature, the PERF motif, the K-helix and the I-helix, which are essential for catalytic activity [18]. The fact that CYPs are often recruited as versatile catalysts in the biosynthesis of SMs makes these enzymes landmarks in the evolution of species-specific chemical diversity [19].

A well-curated set of CYP genes from a particular species is essential for functional identification of the encoded enzymes. In recent years, genome/transcriptome-wide identification of CYPs from plants has been performed to explore their involvement in metabolic pathways [20–24]. For example, Liao et al. [23] identified 118 full-length and 175 partial CYP genes in *Taxus chinensis* (Rehder & E.H.Wilson) Rehder transcriptomes with the aim to discover candidate genes involved in the biosynthesis of diterpenoids including taxol. Chen et al. [24] found 116 full-length and 135 partial CYP genes in *Salvia miltiorrhiza* Bunge transcriptomes with candidates for terpenoid biosynthesis.

PAs were selected to launch the discovery of structural genes causing SM diversity in our study. So far, the only pathway-specific enzyme of PA biosynthesis that has been identified is homospermidine synthase, which converts spermidine and putrescine into homospermidine, the first specific intermediate in the PA biosynthesis pathway [25]. It is not known how homospermidine is converted to the central PA backbone structure senecionine *N*-oxide. Senecionine *N*-oxide undergoes structural transformations in a position-specific and stereoselective manner resulting in the rearrangement of the skeletal structure and oxidative modifications thereof [9]. It was shown that the diversification of PAs in *Jacobaea* species occurs in the shoots while the primary PA senecionine *N*-oxide is synthesized in the roots [26, 27]. With the exception of senecivernine it was deduced that the PA diversification from senecionine *N*-oxide to other PAs is brought about via specific one- or two-step reactions including epoxidation, hydroxylation, dehydrogenation and/or *O*-acetylation [9, 28]. The enzymes responsible for these processes have not been identified. Candidates for the oxidative reactions are members of the CYP family. A comprehensive study and comparison of CYPs between different *Jacobaea* species will be beneficial to identify potential CYP candidates involved in PA biosynthesis.

We have established de novo transcriptome assemblies for *J. vulgaris* and *J. aquatica* and established comprehensive information on CYP families. These two closely related species have been well studied for their PA contrasts [7, 29], but limited genomic or transcriptomic information is available. We first identified putative full-length CYPs classified into different CYP families and extracted the conserved motifs. Furthermore, we investigated the potential involvement of these CYPs in various metabolic pathways based on the KEGG database. We subsequently performed phylogenetic analyses of the largest CYP families in *Jacobaea* species and two other species from the Asteraceae using the CYPs from *Arabidopsis thaliana* (L.) Heynh. as an outgroup to explore relatedness and evolution of CYPs across five species.

## Results

### Transcriptome sequencing and de novo assembly

The purpose of this study was to obtain systematic information of CYPs in *Jacobaea* species, which facilitates further exploration of possible functions in PA metabolism. In total, two sets of samples were obtained for both *J. vulgaris* (Jv1 and Jv2) and *J. aquatica* (Ja1 and Ja2). After removal of adaptor sequences, ambiguous reads and low-quality reads (Q < 30), paired-end clean reads were further processed. The trimmed reads obtained in this study have been deposited in the NCBI SRA database (accession numbers: SRR10013580-SRR10013584 under the BioProject PRJNA561604).

For each of the four sets, more than 20 million cleaned up paired-end reads were used for the de novo assembly with Trinity (Table 1). The resulting assemblies of Jv1, Jv2, Ja1 and Ja2 yielded equal amounts of transcripts containing 152,286, 142,213, 118,936, 130,365 transcripts with average lengths of 936, 1132, 1082 and 1062 nucleotides respectively. To evaluate the qualities of the assembled transcripts, all reads were realigned back to the assemblies using Bowtie2 [30], and we found that between 83 to 91% of reads were mapped back as proper pairs (Table 1). This showed that these assemblies were well-qualified for further mining of CYP genes as our mapping rates were well above the required value of 70–80%.

**Table 1:** Summary of Illumina sequencing and assemblies for two *J. vulgaris* and two *J. aquatica* sets

Sets ^a^	Total paired-end clean reads	Total assembled trinity transcripts	Transcript length range (nt ^b^)	GC content (%)	Contig N50 ^c^ (nt)	Average contig length (nt)	Reads mapped ^d^ (%)
Jv1*	19,725,242	152,286	301–13,238	39.37	1253	936	84.69
Jv2	36,359,675	142,213	301–13,269	39.31	1530	1132	83.25
Ja1*	20,306,518	118,936	301–15,708	39.27	1461	1082	91.57
Ja2	27,505,944	130,365	301–13,309	41.23	1441	1062	87.41

^a^ Jv1 consisted of the pooled shoots and roots of 59 individuals of *J. vulgaris*. Jv2 was composed of shoots from five tissue culture derived plants of *J. vulgaris* treated with MeJA and five mock treated individuals. Ja1 and Ja2 were derived from the same seven individuals of *J. aquatica*, of which roots were included in Ja1 but not in Ja2

^b^ nt: nucleotide

^c^ Contig N50: length such that sequence contigs of this length or longer include half the bases of the Trinity assembly

^d^ Reads mapped: the percentage of properly paired reads mapped back to the Trinity transcriptome assembly by Bowtie2

* cDNA library was normalized before sequencing

### Functional annotation and structural analysis

The Trinotate annotation results of the four de novo assembled transcriptomes can be found in annotation reports (Additional files 1, 2, 3, 4). In total, 28,192 (43.8%), 27,255 (56.3%), 23,023 (48.5%), 22,077 (37.9%) Trinity ‘genes’ were annotated in the GO database for Jv1, Jv2, Ja1 and Ja2, respectively. The percentages of predicted gene functions for the four *Jacobaea* sets were similarly distributed among different functional categories. The MeJA treated set (Ja2) showed a slightly lower percentage in the category “cellular component” but higher percentages in the categories “molecular function” and “biological process” than the other three *Jacobaea* sets (Additional file 5: Fig. S1).

The InDels and structural variants including insertions, deletions, single nucleotide variants (SNVs) and multi-nucleotide variants (MNVs) were detected based on mutual read mapping for the four *Jacobaea* transcriptomes separately. The results are shown in Additional files 1, 2, 3, 4. In addition, the transcripts obtained from each assembly were mined for their simple sequence repeats (SSRs). In total, 13,979, 14,921, 13,337 and 12,970 SSR containing sequences were identified for Jv1, Jv2, Ja1 and Ja2, respectively. The most abundant repeat type was dinucleotides followed by trinucleotides for all assemblies (Additional files 1, 2, 3, 4).

### Identification and classification of CYPs

A total of 221 full-length (Additional file 6: Table S1) and 323 partial CYP genes were identified in *J. vulgaris*, and a total of 157 full-length (Additional file 7: Table S2) with 247 partial CYP genes were identified in *J. aquatica*, respectively. All full-length CYPs were classified and named by Prof. Dr. David R. Nelson. The 221 full-length CYPs of *J. vulgaris* were divided into eight clans and 38 families (17 A-type families, 21 non-A-type families), while the 157 full-length CYPs of *J. aquatica* were divided into eight clans including 35 families (16 A-type families, 19 non-A-type families) (Table 2). Around half of the full-length CYP sequences of both *J. vulgaris* (53.8%) and *J. aquatica* (46.4%) were assigned to CYP71, CYP706, CYP76, CYP72, CYP82 and CYP93 families, of which only CYP72 is non-A-type. Compared with *J. vulgaris*, for *J. aquatica* less full-length CYPs were detected, which might be caused by the lower number of genotypes and the lower amount of reads in the *J. aquatica* samples. However, the proportional distributions of full-length CYPs were similar not only in each CYP clan (Chi-square = 1.6, Df = 8, NS), but also within each CYP family (Chi-square = 18.6, Df = 37, NS) of the two *Jacobaea* species (Table 2).

**Table 2:** Distribution of full-length CYP genes from five species over CYP clans and families

clan	family	Jv^a^	Ja	Ha*	Ls*	At*
51	51	3	4	1	1	2
71	71	41	21	85	74	50
	73	2	4	3	2	1
	75	1	1	3	2	1
	76	14	12	30	25	8
	77	2	2	3	4	5
	78	5	5	8	7	6
	79	1	1	12	6	7
	80	1	0	10	5	0
	81	9	2	32	18	17
	82	11	15	26	32	5
	83	0	0	0	0	2
	84	4	3	7	2	2
	89	3	3	11	5	7
	92	5	2	2	4	0
	93	12	5	7	6	1
	98	5	6	2	2	3
	701	3	2	5	3	1
	703	0	0	1	1	1
	705	0	0	0	0	25
	706	25	12	26	27	7
	712	0	0	0	0	2
	736	0	0	2	5	0
72	72	16	13	40	25	9
	714	2	2	1	1	2
	715	0	0	1	1	1
	721	1	0	4	2	1
	734	0	0	3	2	1
	735	1	1	1	1	2
	749	7	4	6	4	0
74	74	7	4	6	7	2
85	85	1	1	2	1	2
	87	0	0	8	2	1
	88	1	0	1	2	2
	90	5	4	6	7	4
	702	0	0	0	0	6
	707	4	2	9	6	4
	708	0	0	0	0	4
	709	0	0	0	0	3
	716	2	3	24	12	2
	718	0	0	1	1	1
	720	1	1	1	1	1
	722	1	1	1	2	1
	724	0	0	1	1	1
	728	0	0	6	4	0
	729	0	0	1	6	0
	733	0	0	1	1	0
86	86	6	5	9	7	11
	94	2	1	13	10	6
	96	3	3	15	16	13
	704	9	7	18	16	3
97	97	3	3	3	3	3
710	710	1	1	2	1	4
711	711	1	1	2	1	1
total		221	157	462	374	244

^a^*J. vulgaris* (Jv), *J. aquatica* (Ja), *H. annuus* (Ha), *L. sativa* (Ls) and *A. thaliana* (At)

* The numbers of detected full-length CYPs from the transcriptomes of *J. vulgaris* and *J. aquatica* in each clan and family were compared with those from the genomes of three other plant species

We compared the numbers of the detected full-length CYPs of *J. vulgaris* and *J. aquatica* with three other plant species, i.e., *Helianthus annuus* L., *Lactuca sativa* L. and *A. thaliana*. (Table 2). Roughly, the four species of the Asteraceae (*J. vulgaris* 544 (221 full-length and 323 partial CYPs), *J. aquatica* 404 (157 full-length and 247 partial CYP), *H. annuus* 462, *L. sativa* 374) contained more CYP genes than *A. thaliana* (244). It indicates an expansion and functional diversification of CYP genes encoding metabolic pathways in the Asteraceae during evolution and genome duplications.

Overall, the distributions of CYPs among different CYP clans over the five species (Table 2) were comparable (Chi-square = 42.0, Df = 32, NS). However, the distributions among different CYP families were significantly different (Chi-square = 466.7, Df = 212, *P* < 0.001). Numbers of CYPs in single-family CYP clans (CYP51, CYP74, CYP97, CYP710, CYP711) were fairly consistent (Chi-square = 11.2, Df = 16, NS). The significant difference was caused by multiple-family clans (CYP71, CYP72, CYP85, CYP86) which parallel land plant evolution [31] and which have expanded dramatically (Chi-square = 445.6, Df = 192, *P* < 0.001). In accordance with the statement of Nelson and Werck-Reichhart [31], the youngest clan, the CYP71 clan (A-type), was dominant in all five species, of which the CYP71 family possessed the largest numbers of CYPs over all five species. Within the Asteraceae family, ten CYP families were absent in *Jacobaea* species compared with *H. annuus* and *L. sativa*, including CYP703, CYP736, CYP715, CYP734, CYP87, CYP718, CYP724, CYP728, CYP729 and CYP733. Without further information, it is difficult to infer whether the absence/presence is an evolutionary consequence or just due to the unavailability of full-length transcripts in the transcriptomes of *Jacobaea*.

### Characterization of CYP proteins

**Fig. 1:**
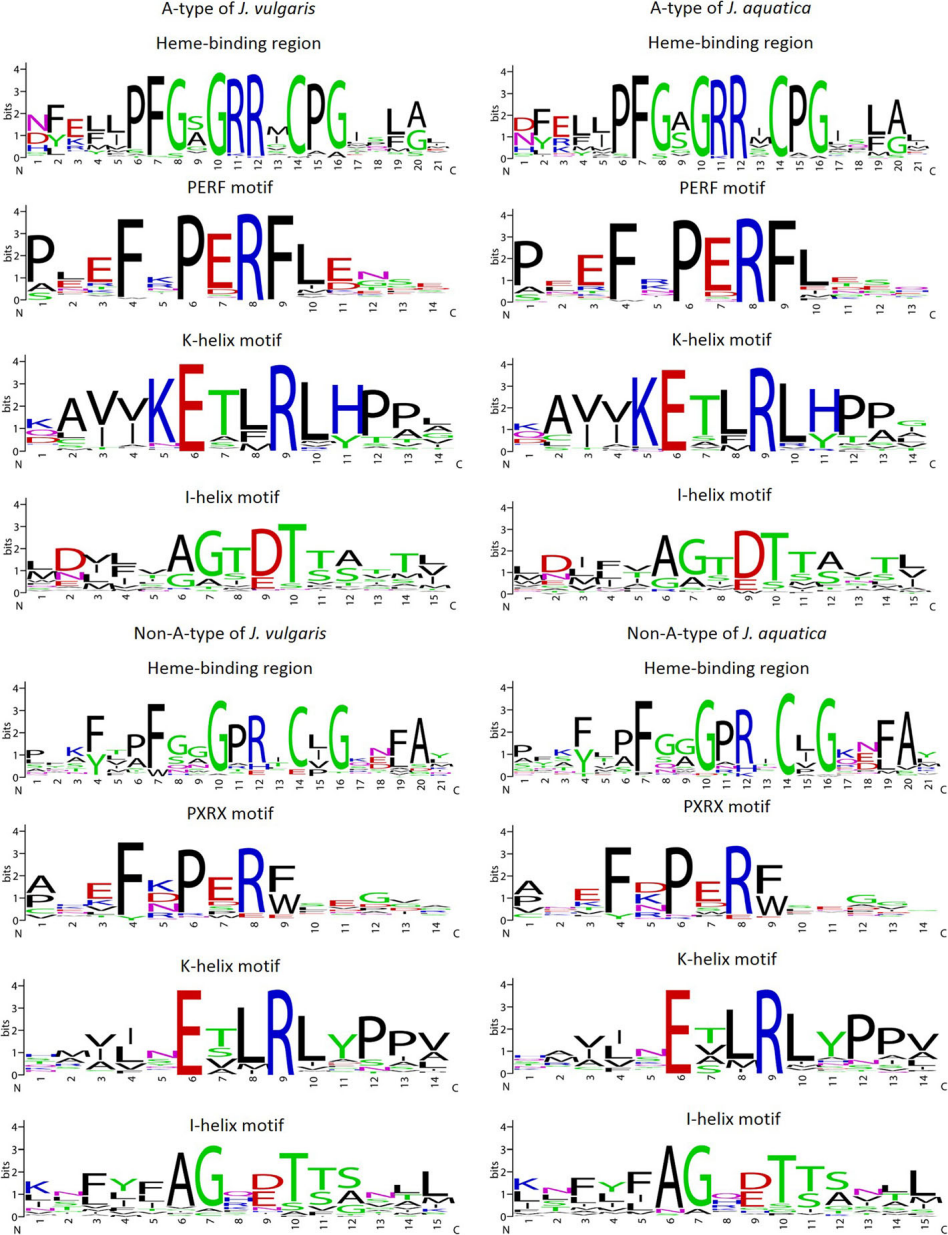
Weblogos of typical conserved motifs identified in the full-length CYP450s of two *Jacobaea* species. CYPs are divided as A-type (the upper figure) and non-A-type (the lower figure) from *J. vulgaris* (left) and *J. aquatica* (right). The names of the motifs are shown above each logo. The bit score indicates the information content for each position in the sequence

The lengths of 221 full-length proteins of *J. vulgaris* (Additional file 6: Table S1) ranged from 460 to 601 amino acids, with an average length of 509 amino acids, and the lengths of 157 full-length proteins of *J. aquatica* (Additional file 7: Table S2) varied from 464 to 601 amino acids with an average length of 511 amino acids. The sequence logos of the four typical conserved motifs including the heme-binding region, the PERF motif, the K-helix region and the I-helix region were extracted (Fig. 1). The consensus sequences of the motifs of *J. vulgaris* and *J. aquatica* were highly similar and also showed high similarities to other plant species [22–24] for both A-type and non-A-type CYP proteins. Furthermore, the differences of signatures of typical motifs (i.e., the heme-binding region, the PERF and the I-helix) between A-type and non-A-type CYPs were also similar to those of other species. The consensus sequence of the heme-binding region of A-type CYPs was “PFGxGRRxCP”, whereas “xFxxGxRxCxG” was found in non-A-type CYPs. The F, G and C residues are conserved in all plant P450s, where the C residue is universally conserved in all P450s across kingdoms and coordinates the iron in the heme [22–24]. For the PERF motif, A-type CYPs displayed the consensus “FxPERF” while non-A-type CYPs showed “FxPxRx”, both with one additional highly conserved “F” which exists in the majority of CYPs [22–24]. The I-helix motifs of A-type and non-A-type CYPs were “AGxDT” and “AGx [D/E]TT”, respectively. The consensus of the ExxR motif of A-type CYPs accorded with that of non-A-type CYPs [22–24]. In line with previous studies [22–24], the results confirmed that plant CYP proteins share well-conserved motifs including the heme-binding signature, the PERF motif, the K-helix and the I-helix, which are essential for catalytic activity [18].

### KEGG pathway analysis of *Jacobaea* CYPs

**Fig. 2:**
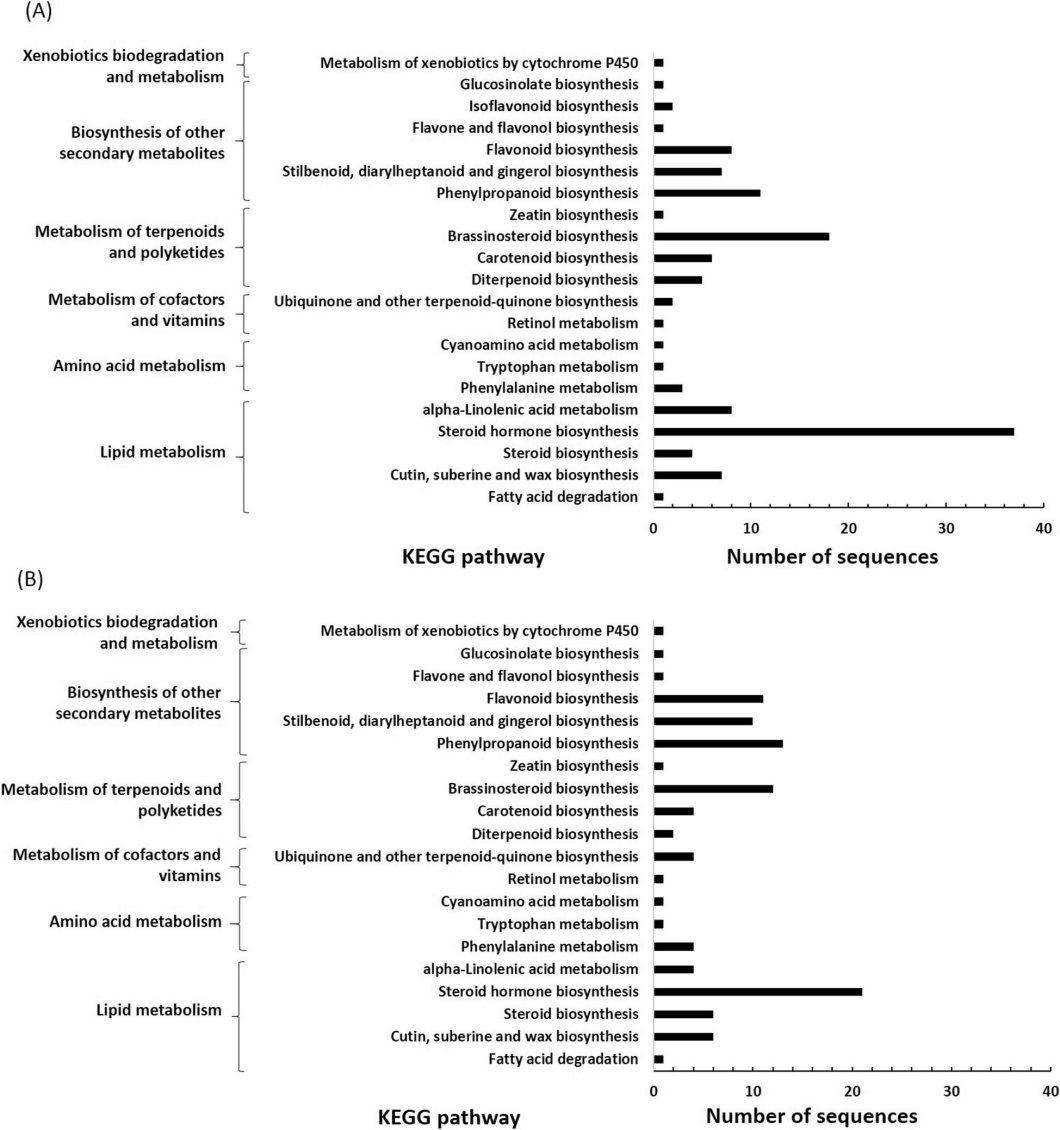
KEGG pathway analysis of predicted CYP450s in two *Jacobaea* species. **a***J. vulgaris*. **b***J. aquatica*. The numbers of CYP450 genes involved in the corresponding metabolic processes are shown

KEGG pathway-based analysis was performed to understand the potential involvement of CYPs in various biosynthetic pathways. Hundred twenty four of the 221 (56.1%) full-length CYPs of *J. vulgaris* were designated to 37 KEGG Ortholog (KO) hierarchies (Additional file 6: Table S1), which were distributed over 21 KEGG pathways (Fig. 2a). For *J. aquatica* 91 out of 157 (58.0%) full-length CYPs were appointed to 33 KO catalogs (Additional file 7: Table S2) covering 20 KEGG pathways (Fig. 2b). In the class of “biosynthesis of other secondary metabolites”, 21 CYPs were assigned to be involved in the biosynthesis of phenylpropanoids (K00487, K09754, K09755), stilbenoids, diarylheptanoids and gingerols (K00487, K09754), flavonoids (K00487, K05280, K09754), flavones and flavonols (K05280), isoflavonoids (K13260) and/or glucosinolates (K12153) for both *Jacobaea* species, of which some genes were assigned to more than one KEGG pathway. All these SM related CYPs belonged to the CYP71 clan. No genes were found to be involved in alkaloid biosynthesis. This does not necessarily mean that they are not involved in alkaloid biosynthesis because this may result from the fact that no information about PA biosynthetic genes is available in the KEGG database yet.

### Phylogenetic analyses

**Fig. 3:**
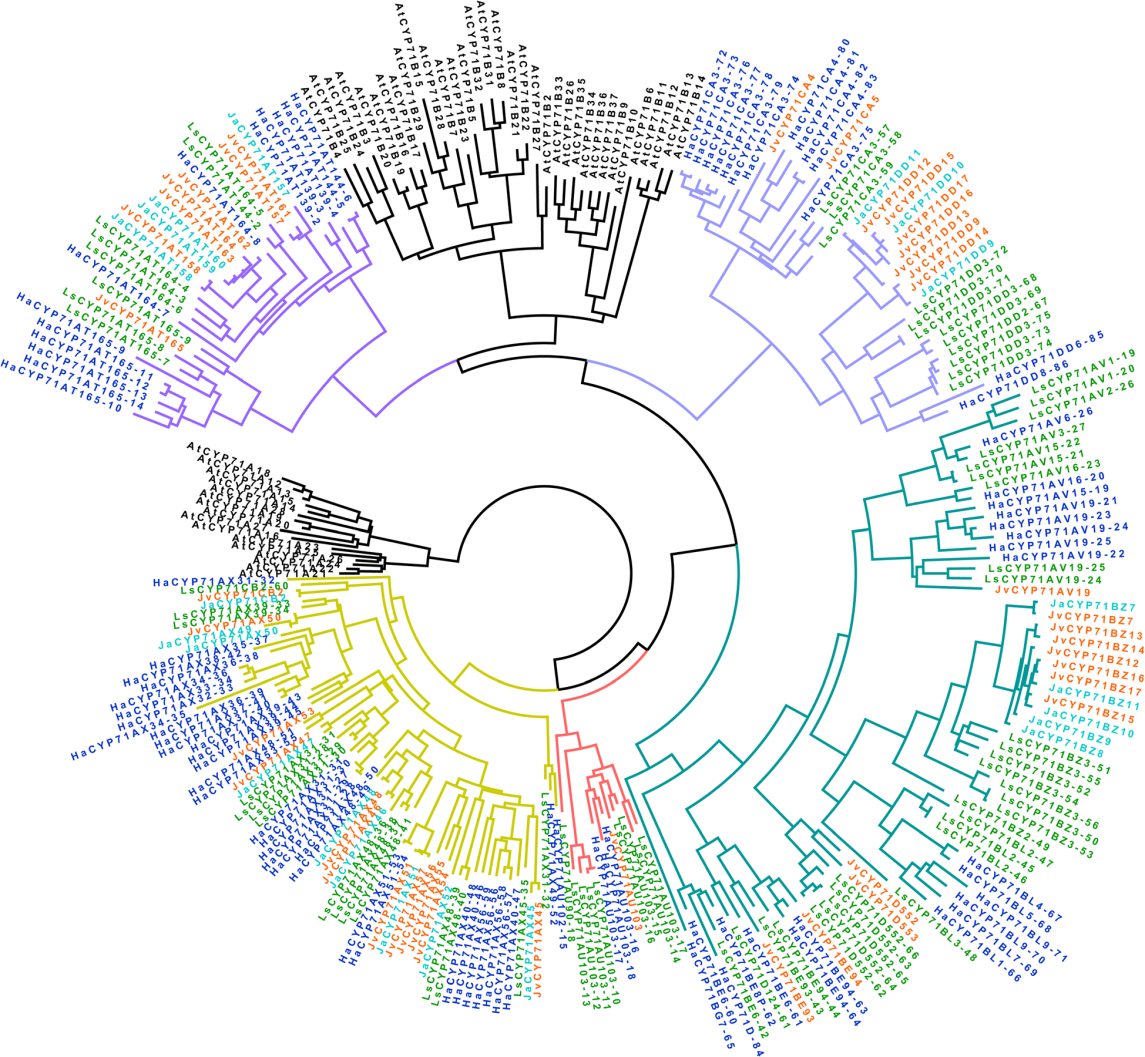
Phylogenetic tree of CYP71 family from 5 species inferred with the maximum likelihood method. CYP450s are color coded for different species: *J. vulgaris* (orange), *J. aquatica* (light blue), *H. annuus* (dark blue), *L. sativa* (green), *A. thaliana* (black). The branches of the five clades of the Asteraceae are color highlighted. The names of CYP450s of *H. annuus* and *L. sativa* were tentatively coded without nomenclature. *A. thaliana* was used as the outgroup

Comparative sequence analysis based on an evolutionary perspective can improve functional prediction [32]. Therefore, we performed phylogenetic analyses using the maximum likelihood (ML) method for the largest six families in *Jacobeae* species, namely, CYP71, CYP76, CYP706, CYP93, CYP82 and CYP72, based on their amino acid sequences (Fig. 3; Additional files 8, 9, 10, 11, 12: Fig. S2-S6). Functional divergence frequently accompanies gene duplication, which was confirmed by our study. Lineage-specific expansion of CYPs was observed overall (Fig. 3; Additional files 8, 9, 10, 11, 12: Figure S2-S6). In all phylogenetic trees, the CYPs from the same species tended to be clustered together, resulting in many lineage-specific subfamilies and/or clades. In most CYP families, CYPs were not equally distributed in different species, suggesting that gene duplication events happened after species divergence. Only within the *Jacobaea* species we observed that often a clade was present with a *J. vulgaris* and a *J. aquatica* CYP. Taking the CYP71 family as example, the CYPs of *A. thaliana* fell into two clades, whereas the CYPs of the Asteraceae species were divided into five distinct clades (Fig. 3). Notably, the speed of evolution of CYPs within the Asteraceae has been very fast resulting in species-specific CYPs. Particularly, the most basal clade of the Asteraceae, the CYP71AX subfamily has expanded dramatically. Even though the distributions of CYPs on the trees were more dispersed compared to those of *A. thaliana*, *Jacobaea* species, *H. annuus* and *L. sativa* all had their own lineage-specific subclades. Only for the closely related species *J. vulgaris* and *J. aquatica*, CYPs were found quite often in pairs, confirming a close relationship in the evolutionary history. For some CYPs of *J. vulgaris* the orthologs were missing in *J. aquatica* (Fig. 3; Additional files 8, 9, 10, 11, 12: Figure S2-S6), which might be caused by less available full-length CYPs of *J. aquatica* in this study or alternatively by the gain or loss of particular CYPs during evolution.

## Discussion

CYPs have an essential function in contributing to chemical diversity that is the landmark of plants [31]. However, as the largest family of enzymes engaged in primary and secondary metabolism and having a fast evolution, CYPs are notorious for their difficulty in classification and nomenclature, which hinders the study of these genes. In the current study, well-curated sets of CYPs with standard nomenclature were obtained for *J. vulgaris* and *J. aquatica*, which is vital for the functional characterization and comparison of these genes. In total, 221 and 157 full-length CYP genes were identified, classified and named from transcriptomes of *J. vulgaris* and *J. aquatica*, respectively.

KEGG pathway-based annotation was performed for all full-length CYPs, and no CYPs were designated to alkaloid biosynthetic pathways. Empirically, CYPs from the same family/subfamily often catalyze similar/related reactions [31]. For example, the CYPs involved in the main reactions of benzylisoquinoline alkaloid diversity include the CYP80 family (CYP80A1, CYP80B3, CYP80G2), the CYP719 family (CYP719A20, CYP719A21, CYP719A25, CYP719B1) and the CYP82 family (CYP82Y1, CYP82Y2, CYP82N4, CYP82X1, CYP82X2) [33]. Nonetheless, consecutive steps in the same alkaloid pathways can be also catalyzed by CYPs from divergent families [31]. For instance, some of the functionally characterized CYPs involved in the monoterpenoid indole alkaloid pathway in *Catharanthus roseus* (L.) G. Don are from different families: CYP71D2, CYP72A1, CYP76B6 [34–37]. Alkaloids are highly species-specific SMs which are characterized by a vast structural diversity. Identifying a CYP catalyzing a particular biosynthetic step is challenging because of the homology shared by CYP proteins and the lack of correlation between primary structure and catalytic function [38], especially since no CYPs involved in PA metabolism have been reported.

CYPs are an excellent reporter of plant evolution, especially in the evolution and role of plant metabolism. An evolutionary approach using phylogenetic trees could be beneficial to CYP function prediction [31]. The diversification of CYPs had a significant biochemical impact on the emergence of new metabolic pathways during the evolutionary process of land plants [39]. In the phylogenetic analyses of the most abundant CYP families of *Jacobaea*, a fast evolution of CYPs was observed resulting in lineage-specific expansion. Notably, CYPs do not always follow the pattern in which *H. annuus* showed a closer phylogenetic relatedness to *Jacobaea* species than *L. sativa* as indicated by Compositae metatrees [40], especially for CYPs in the CYP71 family. Quite often, CYPs in the CYP71 family of *H. annuus* and *L. sativa* switched phylogenetic closeness to those of *Jacobaea* species on the phylogenetic tree (Fig. 3). This suggests that species patterns in CYPs are present. Gene duplication is thought to be one of the major sources of evolutionary innovation, resulting in divergence in paralogs due to neofunctionalization or sub-functionalization [41, 42]. CYP members in multiple-family clans CYP71, CYP72 and CYP85 have enlarged astonishingly, leading to the difficulty in predicting gene functions. However, those CYPs ending in the same clade/subclade in a phylogenetic tree might indicate association with metabolism of particular classes of compounds or similar reactions on different substrates [31].

During plant evolution, individual genes and gene families have been confronted with selection for copy number via duplications, transpositions, and/or deletions [43]. *J. vulgaris* and *J. aquatica* are two phylogenetically closely related but ecologically distinct species both producing PAs with great inter−/intra-specific diversity in their composition and concentration [7]. The question how CYP genes are gained or lost during evolution among these two species is crucial to understanding their chemical diversity. In our study, more full-length CYPs were obtained in *J. vulgaris* and more often some CYP orthologs of *J. vulgaris* (e.g. CYP71BZ and CYP71DD subfamilies) were missing in *J. aquatica*, (Fig. 3; Additional files 8, 9, 10, 11, 12: Fig. S2-S6). This may be attributed to both whole genome duplication and tandem duplication events [44], especially the latter which is essential for the maintenance of large gene families for expanding and contracting rapidly in response to demand of dynamic environment [45]. However, it is not possible to detect the exact duplication/deletion events occurring among these two *Jacobaea* species with only transcriptome data. The smaller number of CYPs from *J. aquatica* may be also explained by the fact that individual plants used for *J. aquatica* (7) were less than those for *J. vulgaris* (69), leading to less comprehensive CYP expression. Based on our study, it is not possible to appoint CYP candidates involved in PA biosynthesis. Nonetheless, the collection of CYPs in *Jacobaea* species can speed up the exploration of function in following studies. As long as whole genome information of *Jacobaea* species is lacking, 5′ Race and 3′ Race techniques can be employed to obtain a more complete collection of full-length CYPs. The prediction of CYP candidates can be further facilitated by correlating gene expression patterns with PA abundances in plants grown under conditions that generate PA contrasts or in F_2_ offspring segregating for PA profiles.

## Conclusion

Here we detected 221 and 157 full-length CYPs for *J. vulgaris* and *J. aquatica*, respectively. Comparison of CYPs over five species showed strong lineage specific diversification of CYPs, indicating fast evolutionary speed of CYPs within the Asteraceae. Only in the closely related *J. vulgaris* and *J. aquatica*, CYPs were found quite often in pairs, confirming a close relationship in the evolutionary history. No genes were found to be involved in alkaloid biosynthesis against KEGG database. Finally, our study presents the first comprehensive overview of CYPs in *Jacobaea* species, which is beneficial for future exploration of their functions, including possible involvement in PA biosynthesis and PA diversity.

## Methods

### Plant material

Aiming for the most comprehensive CYP gene sets, multiple individuals of both *J. vulgaris* and *J. aquatica* originating from different parts of the distribution ranges (Additional file 13: Table S3) were used for transcriptome sequencing because of the large intraspecies variation in both PA composition and concentration. The seeds of *J. vulgaris* collected from Germany were donated by Hortus Botanicus Leiden, and the seeds of *J. aquatica* from UK were donated by Kew Gardens. All other seeds were collected mostly by Dr. K. Vrieling from different locations (Additional file 13: Table S3). No special permission for seed collection is needed. The identification of the samples was conducted by Dr. K. Vrieling. From both *J. vulgaris* and *J. aquatica* species two sets of samples were obtained (Additional file 13: Table S3). The first *J. vulgaris* set (Jv1) consisted of the pooled shoots and roots of 59 individuals from nine different populations across Europe including two individuals derived from tissue culture and one population from Canada (Additional file 13: Table S3). Set Jv1 was normalized. The second *J. vulgaris* set (Jv2) was composed from multiple individuals, clones, of one genotype that was kept in tissue culture. For the set Jv2, five individuals from tissue culture derived plants of *J. vulgaris* treated with methyl jasmonate (MeJA) and five mock treated individuals were used as control. From both MeJA treated and control plants cDNA libraries were obtained that were sequenced separately. The resulting reads were pooled in silico in the later assembly step. Both *J. aquatica* sets (Ja1 and Ja2) were derived from the same seven individuals pooled from two populations with two individuals originating from tissue culture, of which roots were included in Ja1 but not in Ja2 (Additional file 13: Table S3). Set Ja1 was normalized before sequencing while set Ja2 was not.

For sets Jv1, Ja1 and Ja2, seeds were germinated on the surface of wet potting soil covered by plastic bags and the seedlings were transferred into 9 × 9 × 10 cm pots filled with 50% sandy soil (collected from Meijendel), 50% potting soil (Slingerland Potgrond, Zoeterwoude, The Netherlands) and 1.5 g/L Osmocote slow release fertilizer (Scott, Scotts Miracle-Gro, Marysville, Ohio, USA; N: P: K = 15: 9: 11). Tissue cultured plants of *J. vulgaris* and *J. aquatica* were propagated on Murashige and Skoog (MS) medium with 0.44 mM benzylaminopurine. To induce roots plants were transferred to MS medium without hormones for 2 weeks. After rooting plants were transferred to pots filled with the soil mixture as indicated above. All plants were kept in a climate room for 6 weeks (humidity 70%, light 16 h at 20 °C, dark 8 h at 20 °C). Then the plants were separated into shoots and roots, and roots were rinsed with water. Two to three fully grown leaves and ¼ of roots from each plant were wrapped in aluminum foil and flash frozen in liquid nitrogen, respectively. Afterwards all samples were separately ground into powder with liquid nitrogen. Shoot powder was mixed with root powder in a ratio of 3:1 for each plant, and then identical amounts of powder from each individual were pooled for Jv1 and Ja1, respectively, whereas only powdered shoots were pooled for Ja2. All powdered materials were stored at − 80 °C until RNA extraction.

For set Jv2, replicate *J. vulgaris* tissue culture plants were kept on MS medium with agar for 2 weeks after propagation in a climate room (50% humidity, light 16 h at 20 °C, dark 8 h at 20 °C). One hundred microliters of MeJA (Sigma-Aldrich) dissolved in 10% ethanol solution (4.5 mmol/L) was added to the surface of medium, reaching a final concentration of 90 μmol/L after diffusion in each tube, while the same volume of 10% ethanol was added to the control group under axenic condition. Shoots of five biological replicates collected at 8 days after the treatment were pooled and ground into fine powder for both induced and control groups, respectively. All powder was stored at − 80 °C until RNA extraction.

### RNA isolation, normalization and transcriptome sequencing

Total RNA was extracted with the NucleoSpin® RNA Plant-Macherey-Nagel kit for five samples, namely Jv1, MeJA induced group of Jv2, control group of Jv2, Ja1 and Ja2. The RNA integrity Number (RIN) and RNA concentration were assessed using the Agilent 2100 Bioanalyzer. Strand specific RNAseq libraries were generated using the method described by [46] with minor modifications by the Leiden Genome Technology Center. In short, polyA+ mRNA was isolated from 1 μg of total RNA using oligo-dT Dynabeads (LifeTech 61,002) and fragmented to 150–200 nucleotides in first strand buffer for 3 min at 94 °C. Random hexamer primed first strand was generated in presence of dATP, dGTP, dCTP and dTTP. dUTP was used to tag the second strand instead of dTTP. Subsequent steps to construct the sequencing libraries were performed with the KAPA HTP Library Preparation Kit for Illumina sequencing with minor modifications. Shortly, after indexed adapter ligation to the dsDNA fragments, the libraries were treated with USER enzyme (NEB M5505L) in order to digest the second strand derived fragments. Pre-amplified library yields were quantified on an Agilent high sensitivity chip. Two of four sets (Jv1 and Ja1) were normalized with duplex-specific thermostable nuclease (DSN, Evrogen) to remove abundant library molecules aiming at enhancing the gene discovery rate. The protocol was carried out according to the Illumina guidelines for Jv1 and Ja1. After DSN treatment, a second round of PCR was performed. All samples were quantified on an Agilent high sensitivity chip prior to pooling in equimolar amounts and sequencing on a HiSeq2500 with 2 × 126 bp paired-end reads in the Leiden Genome Technology Center.

### De novo assembly and evaluation

After removal of adapter sequences, the qualities of raw reads were checked using FastQC and the bases with low quality (threshold < 30) were cut off by Trimmomatic via the Galaxy platform [47]. The paired-end clean reads were used for assembly. A de novo assembly strategy using the Trinity program [48] with a k-mer size of 32 and the minimum assembled contig length to report set to 300 bp was employed to assemble the four sets (Jv1, Jv2, Ja1 and Ja2). To assess the quality of four assemblies, reads were aligned back to transcriptomes by Bowtie2 [30]. GC content and basic statistics values were calculated using the script imbedded in the Trinity suite.

### Functional annotation and structural analysis

The likely coding regions and open reading frames (ORFs) of transcriptomes were predicted with TransDecoder [49]. The transcriptome functional annotation and analysis was conducted using the Trinotate pipeline [50]. Specifically, the transcripts and the TransDecoder predicted peptides were searched for their homologs against the UniProtKB/Swiss-Prot database using BLASTx and BLASTp, respectively. In addition, protein domains were identified with HMMER program [51] against Pfam database. The presence and location of signal peptide cleavage sites were predicted with the signalP 4.1 server [52], and the prediction of transmembrane helices in proteins were performed using the TMHMM server v.2.0 [53]. Annotation outputs were loaded into the corresponding Trinotate SQLite Database for each transcriptome, and corresponding annotation reports were generated. GO assignments were extracted by using the script “extract_GO_assignments_from_Trinotate_xls.pl” in Trinotate. By utilizing WEGO 2.0 [54] Gene Ontology (GO) annotation results were plotted and compared among four *Jacobaea* sets at the Trinity ‘gene’ level containing a cluster of transcript isoforms.

The combined reads of *J. aquatica* were mapped to the transcriptomes of Jv1 and Jv2 separately, whereas the combined reads of *J. vulgaris* were mapped to the transcriptomes of Ja1 and Ja2 separately. The detection of InDels and structural variants was performed based on the read mapping in CLC Genomics Workbench (version 8.5.1) using default parameters except that the *P*-Value threshold was set to 0.00001. In addition, SNVs and MNVs were detected also based on the abovementioned read mapping using the Basic Variant Detection tool of the Variant Detectors module in CLC Genomics Workbench with default parameters. SSRs were identified using the MISA MicroSatellite identification tool [55], setting a minimum repeat length criteria of six repeated units for dinucleotides, five repeated units for tri, tetra, penta and hexanucleotides, and two SSR were separated by a maximum distance of 100 nucleotides.

### In silico mining of CYP genes

To identify CYP-like contigs from the four transcriptomes, the HMMER program [51] was used to search for homologs by the hidden Markov model against the CYP reference (PF00067) of the Pfam database [56], with an e-value cutoff of 1e-5. The obtained CYP-like contigs from sets Jv1 and Jv2 of *J. vulgaris* were combined and 100% identical transcripts were removed by using the CD-HIT-EST algorithm (version 4.6.8) [57, 58]. For *J. aquatica*, the sample approach was applied to combine CYP-like contigs from sets Ja1 and Ja2.

To obtain additional CYP-like contigs, the reads of *J. vulgaris* were mapped to all CYP-like contigs of *J. aquatica* in CLC Genomics Workbench using the following parameters: mismatch cost 2, insertion cost 3, deletion cost 3, length fraction 0.8, similarity fraction 0.97. The consensus sequences of the mapped reads were retained and assembled with the original CYP-like contigs of *J. vulgaris* in Sequencher (version 5.0), using a minimum match percentage of 97% while minimum overlap was set to 15%. Thereupon, the Sequencher assembly of CYP-like contigs were checked for redundancies using the CD-HIT-EST algorithm with sequence identity of 97% as cutoff. Similarly, to get additional CYP-like contigs for *J. aquatica*, CYP-like contigs of *J. vulgaris* were used as references for read mapping, followed by the same steps afterwards.

The likely coding regions of the resultant CYP-like contigs of both species were predicted by TransDecoder [49]. In order to recognize full-length CYP genes, all the peptide sequences were blasted against NCBI, and the information of blast hits were used to classify CYPs into different clans. Within each clan the alignment of sequences which contain at least 400 amino acids was conducted in MEGA 7 [59] for manual curation of complete coding regions. The putative full-length CYP genes were identified according to the following two criteria: (1) the corresponding proteins starts with amino acid ‘M’ and stops before a stop codon; (2) The aligned regions within each clan cover most of the length in a blast hit to a full-length CYP at the NCBI database, where the highly conserved heme signature is about 50 amino acids from the C-terminus.

### Classification and characterization of *Jacobaea* CYP genes

The final classification and nomenclature of all full-length CYP proteins were carried out by Prof. Dr. David R. Nelson through comparison with references from a well-annotated plant CYP database which includes both published and confidential sequences, following the CYP nomenclature principle [60]. Cutoff values for family, subfamily and allelic variants were 40, 55 and 97% amino acid sequence identity, respectively.

The CYP assemblies were divided into A-type which only comprises the CYP71 clan, and non-A-type which includes all other plant CYP clans. The sequences of A-type and non-A-type were separately submitted to Multiple Expectation Maximization for Motif Elicitation (MEME) to predict motifs and to Motif Alignment and Search Tool (MAST) to discover homologs [61]. The logos of motifs were created using WEBLOGO [62, 63]. Furthermore, the theoretical isoelectric points (PI) and molecular weights (kDa) were predicted by the “Compute pI/Mw tool” on the ExPASy server [64] and the subcellular locations were predicted using the TargetP1.1 server with specificity > 0.95 [65]. KEGG Automatic Annotation Server (KAAS) [66] was used for ortholog assignment and pathway mapping using the SBH (single-directional best hit) method with the BLAST program.

### Phylogenetic analysis

The CYP protein sequences of *H. annuus* [67] and *L. sativa* [68] were retrieved from their transcriptomes using the same approach as aforementioned for *Jacobaea* species based on homologs by the HMM model. Only CYPs longer than 400 amino acids were chosen in this study as the length of the most reliably annotated CYPs of *A. thaliana* ranges from 457 to 594 amino acids without taking pseudogenes into account. All chosen CYP genes were classified based on the best blast hits by Prof. Dr. David R. Nelson. The CYP protein sequences of *A. thaliana* were downloaded from the *Arabidopsis* Cytochrome P450 database [69]. Multiple sequence alignments were performed respectively for putative full-length CYP genes in CYP71, CYP76, CYP706, CYP82, CYP93 and CYP72 families using the MUSCLE module imbedded in the MEGA 7 package [59] using default settings followed by manual editing. Phylogenetic trees were inferred by using the ML method. The trees were obtained with IQ-tree [70, 71] on XSEDE through CIPRES Science Gateway [72]. Bootstrap (BS) search was conducted using standard nonparametric bootstrap with 1000 replicates.

## Supplementary information

**Additional file 1.** Functional annotation and structural analysis of Jv1 transcriptome. The file contains the information of gene functional annotation, InDels, structural variants and simple sequence repeats (SSRs).

**Additional file 2.** Functional annotation and structural analysis of Jv2 transcriptome. The file contains the information of gene functional annotation, InDels, structural variants and simple sequence repeats (SSRs).

**Additional file 3.** Functional annotation and structural analysis of Ja1 transcriptome. The file contains the information of gene functional annotation, InDels, structural variants and simple sequence repeats (SSRs).

**Additional file 4.** Functional annotation and structural analysis of Ja2 transcriptome. The file contains the information of gene functional annotation, InDels, structural variants and simple sequence repeats (SSRs).

**Additional file 5: Figure S1.** WEGO histogram representation of GO classification for transcriptomes of Jv1, Jv2, Ja1 and Ja2. X-axis shows user selected GO terms; left y-axis shows the percentages of genes (number of a particular gene divided by total gene number).

**Additional file 6: Table S1.** List of full-length CYPs of *J. vulgaris* identified in this study. ^a^Cellular location of the protein predicted by TargetP. “C” chloroplast; “S” secreted; “_” any other location; “*” unknown. ^b^not available. ^c^KEGG Orthology.

**Additional file 7: Table S2.** List of full-length CYPs of *J. aquatica* identified in this study. ^a^Cellular location of the protein predicted by TargetP. “C” chloroplast; “S” secreted; “_” any other location; “*” unknown. ^b^not available. ^c^KEGG Orthology.

**Additional file 8: Figure S2.** Phylogenetic tree of the CYP76 family from 5 species inferred with the maximum likelihood method. CYP450s are color coded for different species: *J. vulgaris* (orange), *J. aquatica* (light blue), *H. annuus* (dark blue), *L. sativa* (green), *A. thaliana* (black). The names of CYP450s of *H. annuus* and *L. sativa* were tentatively coded without nomenclature. *A. thaliana* was used as the outgroup.

**Additional file 9: Figure S3.** Phylogenetic tree of the CYP706 family from 5 species inferred with the maximum likelihood method. CYP450s are color coded for different species: *J. vulgaris* (orange), *J. aquatica* (light blue), *H. annuus* (dark blue), *L. sativa* (green), *A. thaliana* (black). The names of CYP450s of *H. annuus* and *L. sativa* were tentatively coded without nomenclature. *A. thaliana* was used as the outgroup.

**Additional file 10: Figure S4.** Phylogenetic tree of the CYP82 family from 5 species inferred with the maximum likelihood method. CYP450s are color coded for different species: *J. vulgaris* (orange), *J. aquatica* (light blue), *H. annuus* (dark blue), *L. sativa* (green), *A. thaliana* (black). The names of CYP450s of *H. annuus* and *L. sativa* were tentatively coded without nomenclature. *A. thaliana* was used as the outgroup.

**Additional file 11: Figure S5.** Phylogenetic tree of the CYP93 family from 5 species inferred with the maximum likelihood method. CYP450s are color coded for different species: *J. vulgaris* (orange), *J. aquatica* (light blue), *H. annuus* (dark blue), *L. sativa* (green), *A. thaliana* (black). The names of CYP450s of *H. annuus* and *L. sativa* were tentatively coded without nomenclature. *A. thaliana* was used as the outgroup.

**Additional file 12: Figure S6.** Phylogenetic tree of the CYP72 family from 5 species inferred with the maximum likelihood method. CYP450s are color coded for different species: *J. vulgaris* (orange), *J. aquatica* (light blue), *H. annuus* (dark blue), *L. sativa* (green), *A. thaliana* (black). The names of CYP450s of *H. annuus* and *L. sativa* were tentatively coded without nomenclature. *A. thaliana* was used as the outgroup.

**Additional file 13: Table S3.** Details of sample sets of *J. vulgaris* and *J. aquatica* for deep sequencing analysis. *cDNA library was normalized with duplex-specific thermostable nuclease to remove abundant library molecules.

## Data Availability

The dataset generated and analyzed during the study are included in this published article and its supplementary information files, or are available from the corresponding authors on reasonable request. The Illumina RNA-sequencing reads are available in the NCBI Sequence Read Archive database (http://www.ncbi.nlm.nih.gov/sra/) under the accession numbers: SRR10013580, SSR 100135581, SRR10013582, SRR10013583, SRR10013584 under the BioProject of PRJNA561604.

## Abbreviations

SM: Secondary metabolite
CYP: Cytochrome P450 monooxygenase
PA: Pyrrolizidine alkaloid
Jv: *Jacobaea vulgaris*
Ja: *Jacobaea aquatica*
Ha: *Helianthus annuus*
Ls: *Lactuca sativa*
At: *Arabidopsis thaliana*
MeJA: Methyl jasmonate
GO: Gene Ontology
SNVs: Single nucleotide variants
MNVs: Multi-nucleotide variants
SSRs: Simple sequence repeats
KO: KEGG Ortholog
DSN: Duplex-specific thermostable nuclease
MS: Murashige and Skoog
HMM: Hidden Markov model
MEME: Multiple Expectation Maximization for Motif Elicitation
MAST: Motif Alignment and Search Tool
KAAS: KEGG Automatic Annotation Server
ML: Maximum likelihood
