# Supplementary figures and images for: Diversity and evolution of cytochrome P450s of Jacobaea vulgaris and Jacobaea aquatica
Source: BMC Plant Biol. 2020 Jul 20;20:342. doi: 10.1186/s12870-020-02532-y (PMC7372880; doi:10.1186/s12870-020-02532-y)

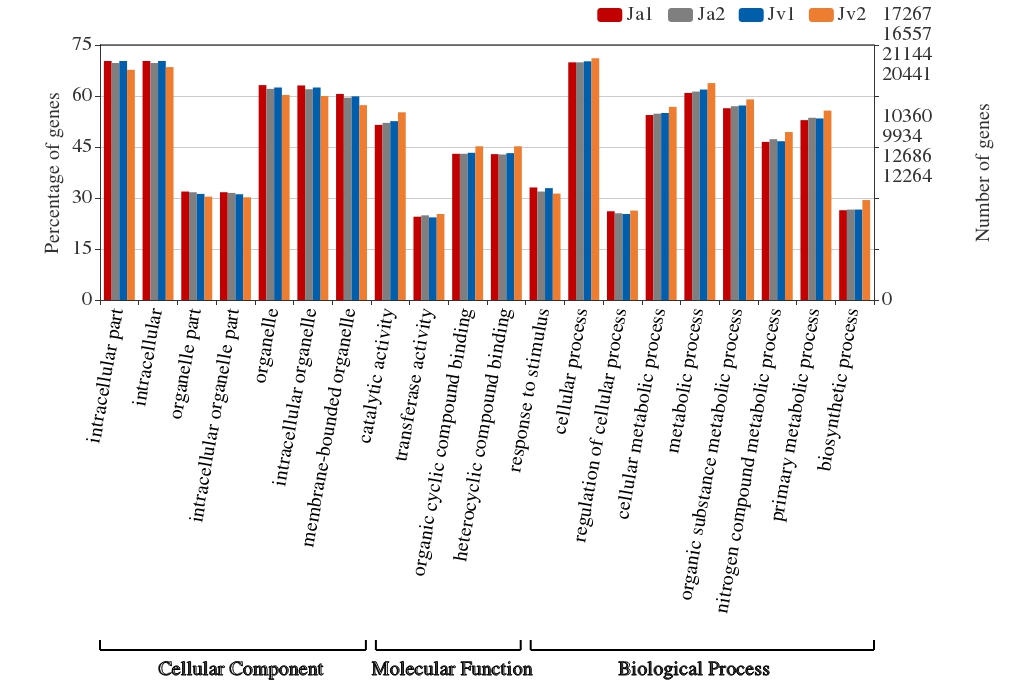

Supplement: Supplementary file 5 — Additional file 5: Figure S1. WEGO histogram representation of GO classification for transcriptomes of Jv1, Jv2, Ja1 and Ja2. X-axis shows user selected GO terms; left y-axis shows the percentages of genes (number of a particular gene divided by total gene number). [file 12870_2020_2532_MOESM5_ESM.jpeg]

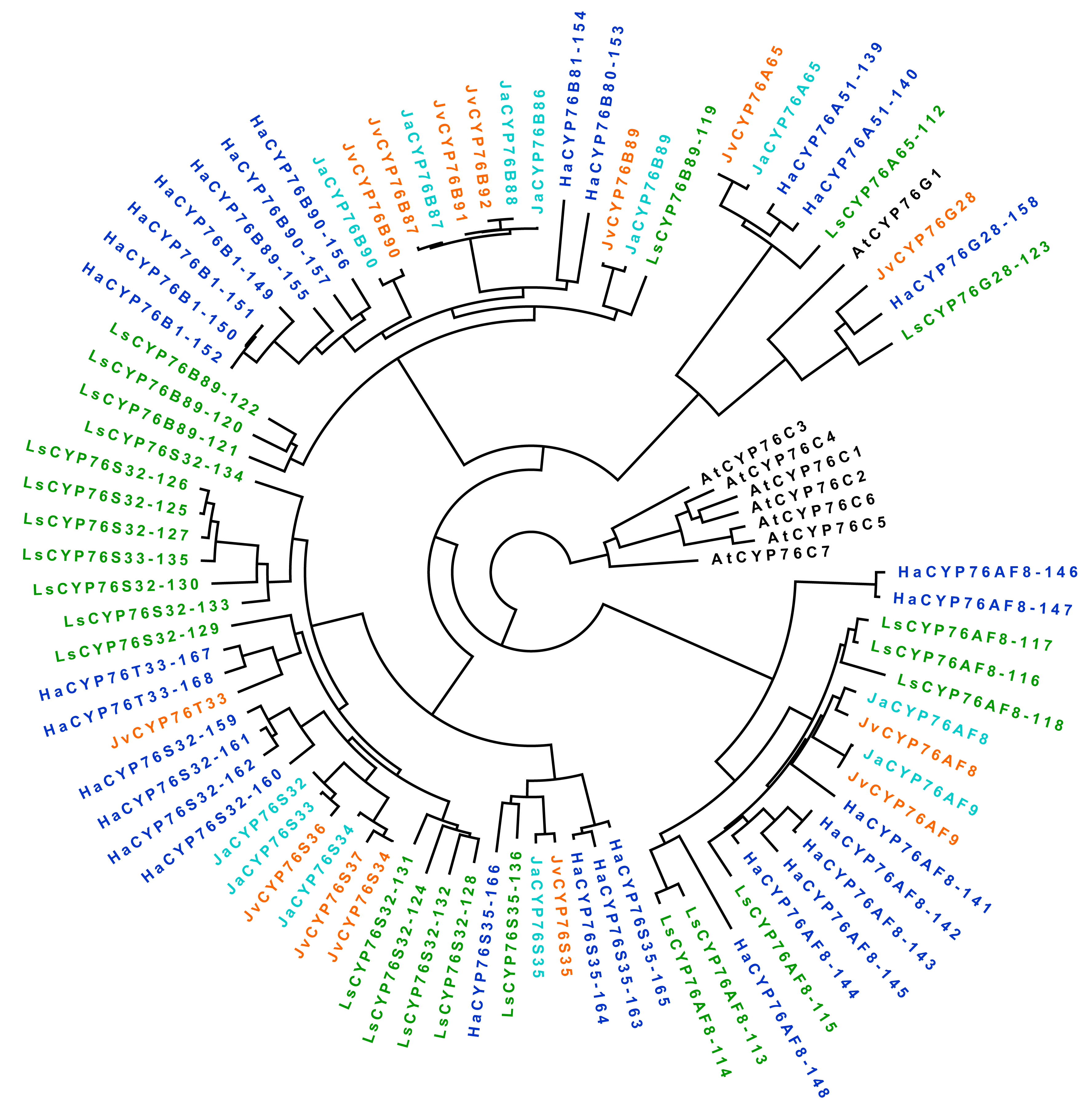

Supplement: Supplementary file 8 — Additional file 8: Figure S2. Phylogenetic tree of the CYP76 family from 5 species inferred with the maximum likelihood method. CYP450s are color coded for different species: J. vulgaris (orange), J. aquatica (light blue), H. annuus (dark blue), L. sativa (green), A. thaliana (black). The names of CYP450s of H. annuus and L. sativa were tentatively coded without nomenclature. A. thaliana was used as the outgroup. [file 12870_2020_2532_MOESM8_ESM.tif]

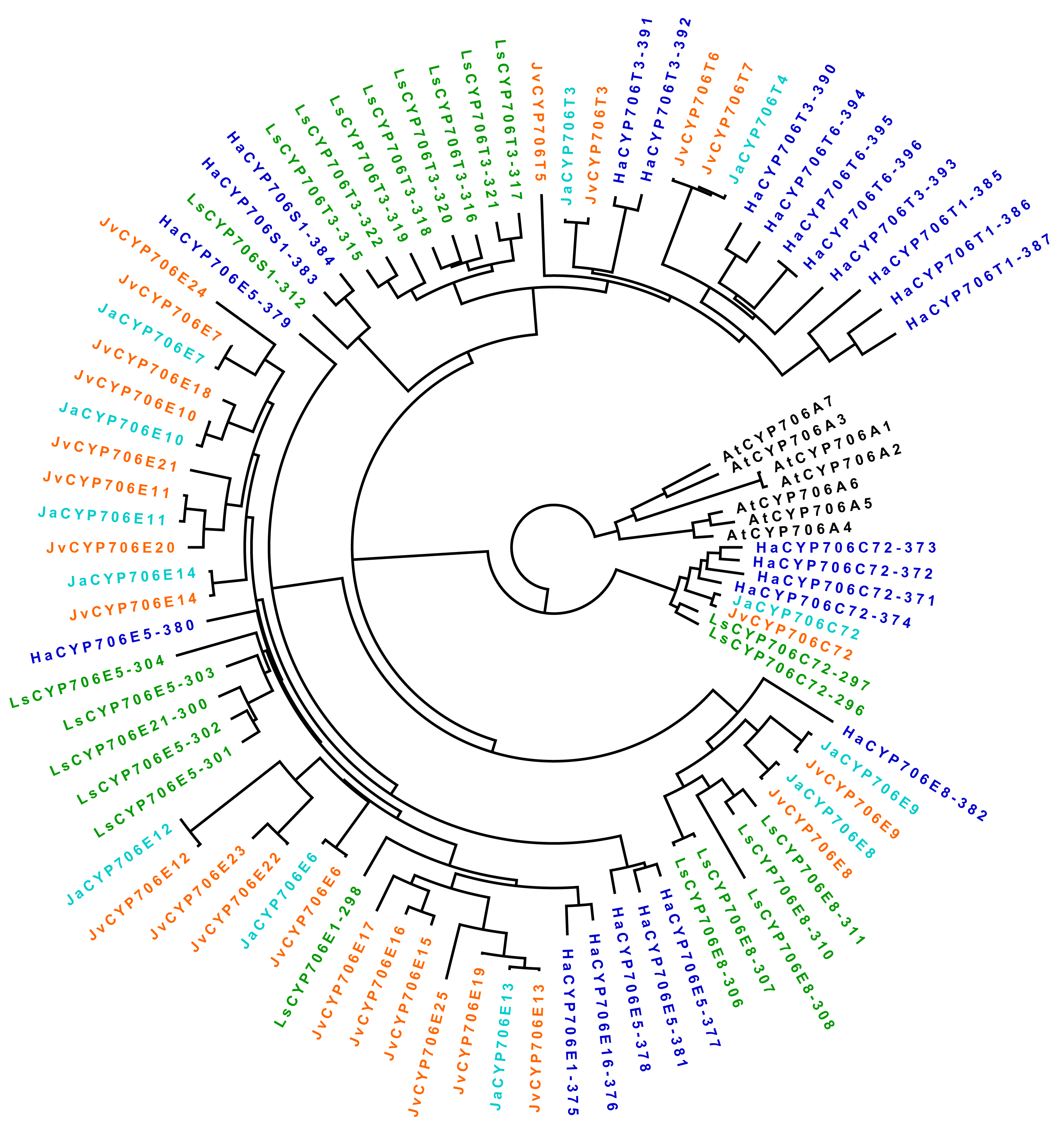

Supplement: Supplementary file 9 — Additional file 9: Figure S3. Phylogenetic tree of the CYP706 family from 5 species inferred with the maximum likelihood method. CYP450s are color coded for different species: J. vulgaris (orange), J. aquatica (light blue), H. annuus (dark blue), L. sativa (green), A. thaliana (black). The names of CYP450s of H. annuus and L. sativa were tentatively coded without nomenclature. A. thaliana was used as the outgroup. [file 12870_2020_2532_MOESM9_ESM.tif]

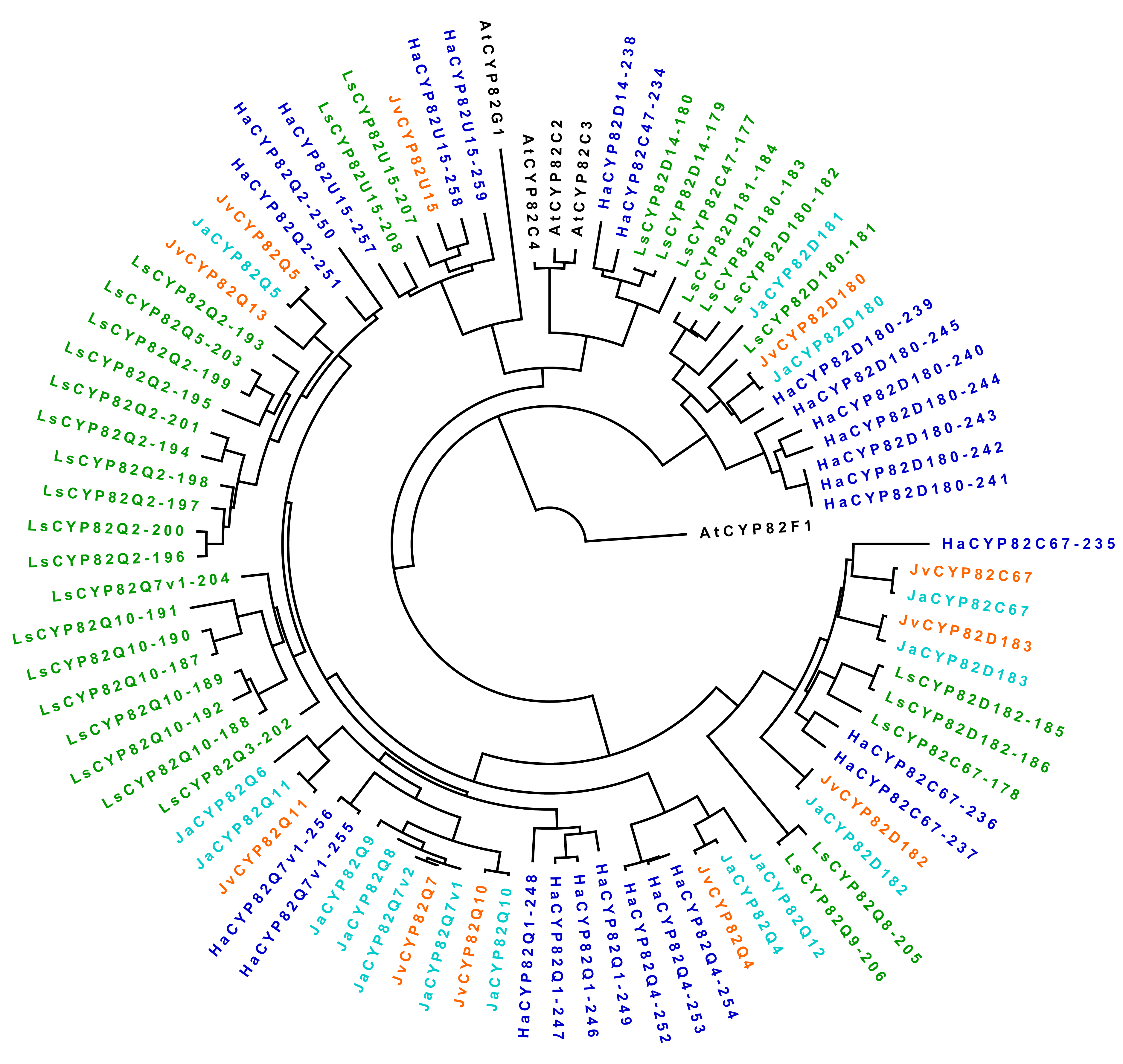

Supplement: Supplementary file 10 — Additional file 10: Figure S4. Phylogenetic tree of the CYP82 family from 5 species inferred with the maximum likelihood method. CYP450s are color coded for different species: J. vulgaris (orange), J. aquatica (light blue), H. annuus (dark blue), L. sativa (green), A. thaliana (black). The names of CYP450s of H. annuus and L. sativa were tentatively coded without nomenclature. A. thaliana was used as the outgroup. [file 12870_2020_2532_MOESM10_ESM.tif]

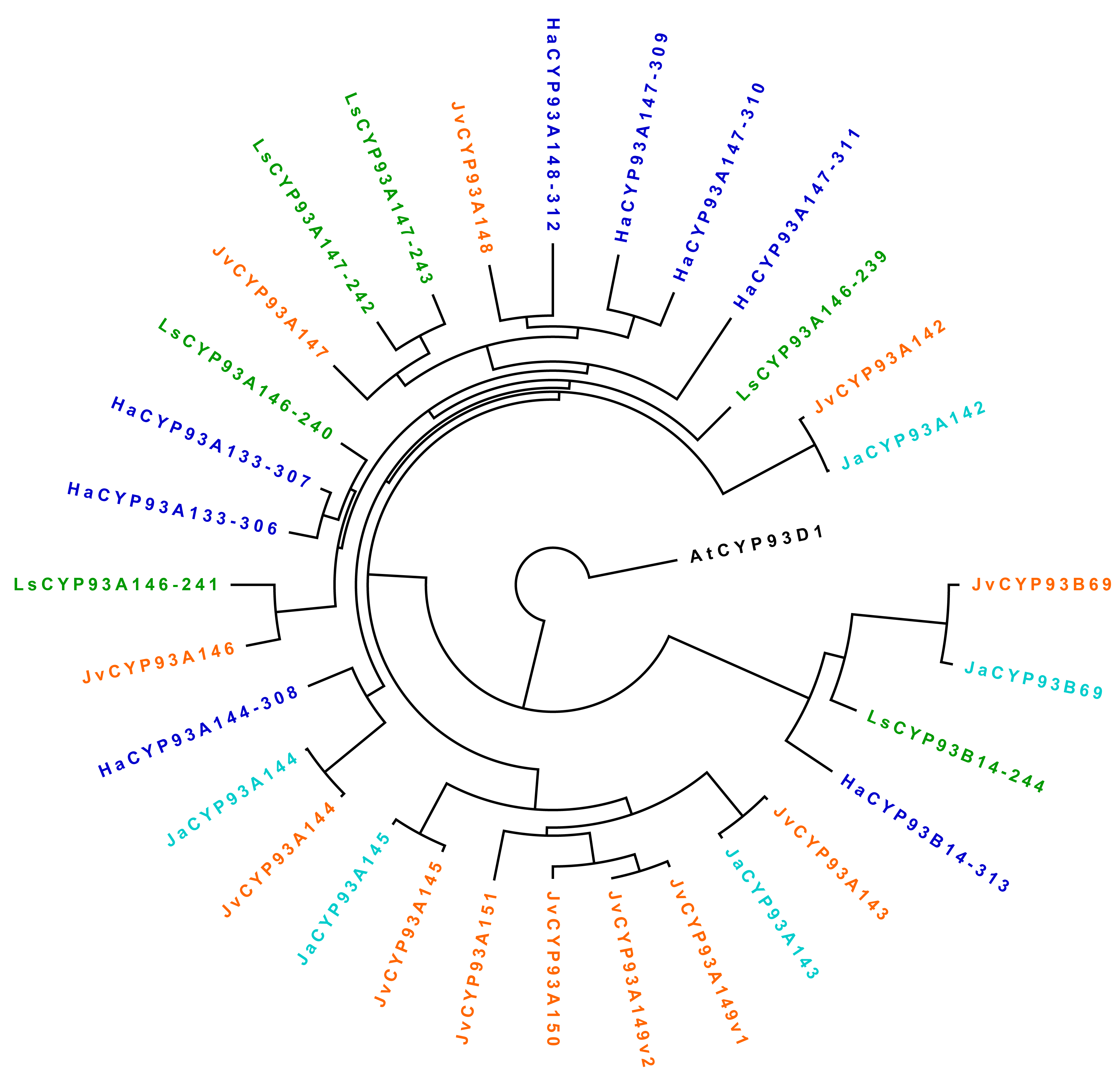

Supplement: Supplementary file 11 — Additional file 11: Figure S5. Phylogenetic tree of the CYP93 family from 5 species inferred with the maximum likelihood method. CYP450s are color coded for different species: J. vulgaris (orange), J. aquatica (light blue), H. annuus (dark blue), L. sativa (green), A. thaliana (black). The names of CYP450s of H. annuus and L. sativa were tentatively coded without nomenclature. A. thaliana was used as the outgroup. [file 12870_2020_2532_MOESM11_ESM.tif]

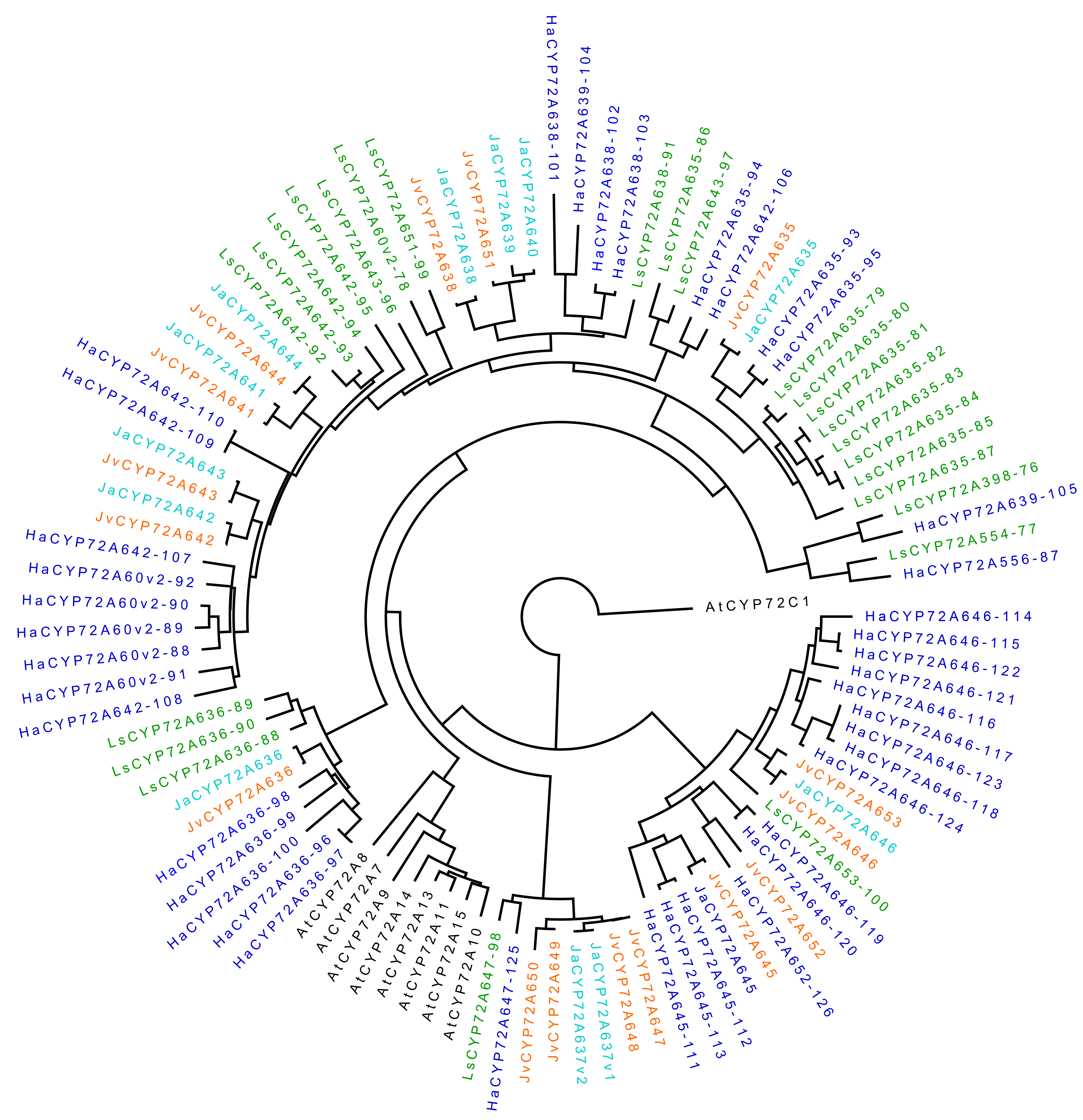

Supplement: Supplementary file 12 — Additional file 12: Figure S6. Phylogenetic tree of the CYP72 family from 5 species inferred with the maximum likelihood method. CYP450s are color coded for different species: J. vulgaris (orange), J. aquatica (light blue), H. annuus (dark blue), L. sativa (green), A. thaliana (black). The names of CYP450s of H. annuus and L. sativa were tentatively coded without nomenclature. A. thaliana was used as the outgroup. [file 12870_2020_2532_MOESM12_ESM.tif]
